# Supplementary material for: A Global Screen for Assembly State Changes of the Mitotic Proteome by SEC-SWATH-MS
Source: Cell Syst. 2020 Feb 26;10(2):133–155.e6. doi: 10.1016/j.cels.2020.01.001 (PMC7042714; doi:10.1016/j.cels.2020.01.001)

Q29RF7 | PDS5A_HUMAN | PDS5A KIAA0648 PDS5 PIG54
Monomer MW [kDa]: 150.83 Monomer expected elution fraction: 37

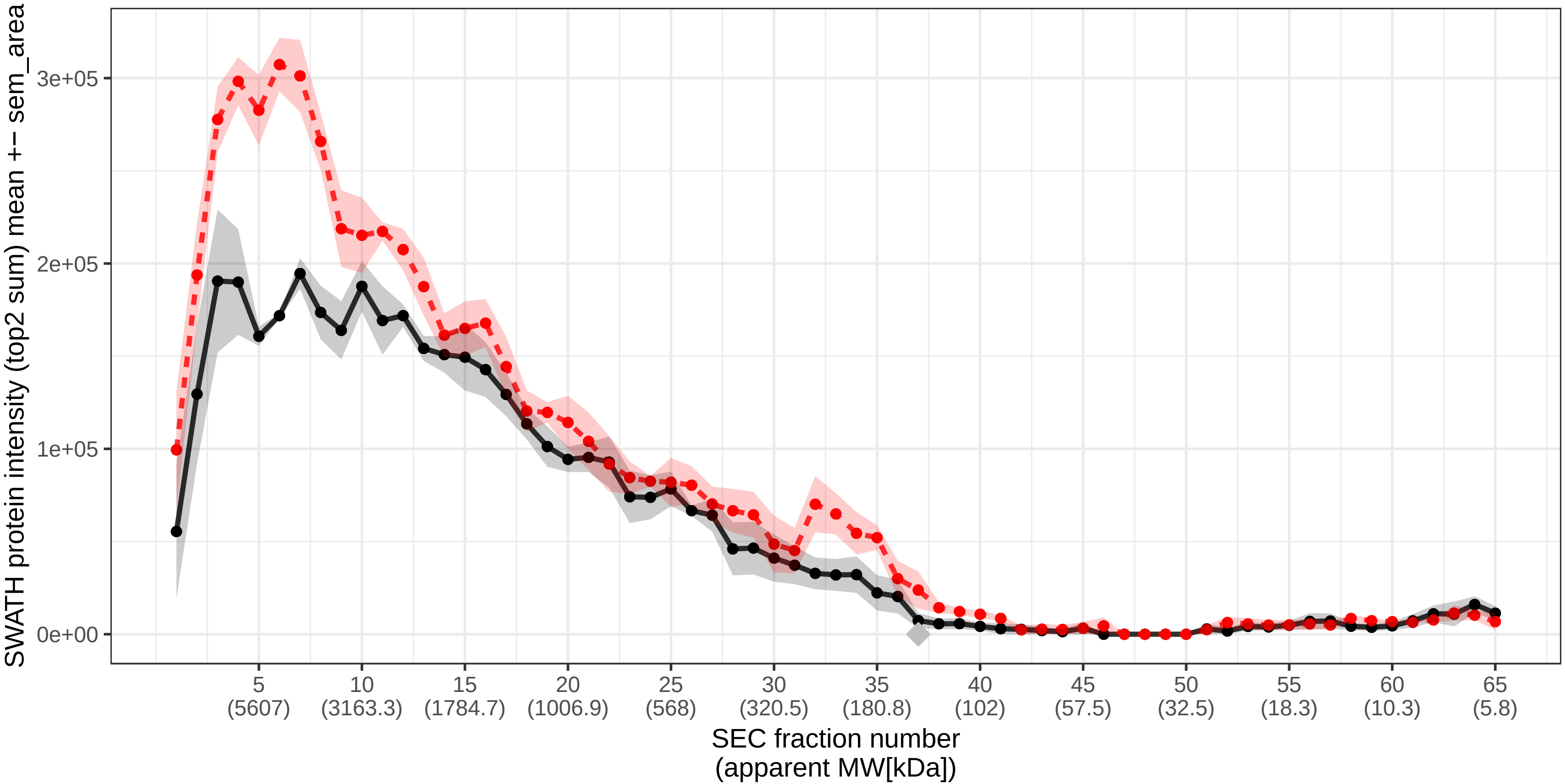

Supplement: Data S1. SEC-SWATH-MS Protein Chromatograms, Related to Figure 1 [file mmc6.zip › SECchrom_Q29RF7_PDS5A_HUMAN_PDS5A_KIAA0648_PDS5_PIG54.pdf]
